# Combined endoscopic and laparoscopic approach for palliative resection of metastatic melanoma of the stomach

**DOI:** 10.1186/1477-7819-4-20

**Published:** 2006-03-30

**Authors:** RS Date, EA Griffiths, SA Pritchard, I McL Welch

**Affiliations:** 1 Department of Gastrointestinal Surgery, South Manchester University Hospital, NHS Trust, South Moor Road, Wythenshawe, Manchester, M23 9LT, UK

## Abstract

**Background:**

Metastatic tumours of the stomach present a clinical dilemma for the surgeon. Palliative surgical resection can alleviate symptoms and prolong survival in selected patients. However, previous studies have used open methods of surgical resection with potentially high morbidity and mortality. We describe the use of laparoscopic wedge resection of the stomach for palliative resection of metastatic melanoma to highlight the benefits of this technique.

**Case presentation:**

A 58 year old male was investigated for iron deficiency anaemia while under treatment for pulmonary metastatic malignant melanoma. An upper gastrointestinal endoscopy revealed a 5 cm diameter ulcer on the anterior wall of the stomach, biopsies from the ulcer confirmed metastatic melanoma. Laparoscopic wedge resection of the stomach lesion was performed without complication.

**Conclusion:**

Laparoscopic approach has many benefits and is useful for the palliative resection of rare tumours of the stomach in order to preserve the quality of life. Its use should be considered in selected patients.

## Background

Gastrointestinal metastases from malignant melanoma (MM) represent a clinical problem. Previous studies have shown that surgical resection offers good symptom palliation, prevents future emergency presentation and prolongs survival [1-3]. However, these patients often only have a median of 6 to 12 months life expectancy [1,3] and offering surgical treatment poses a clinical dilemma. Some previous studies of open surgical resection methods have described high morbidity and mortality rates; 20 and 15% respectively [4]. Although others have described acceptable morbidity and mortality with open surgery [1], laparoscopic surgery is likely to have the potential to further reduce morbidity from these finds of procedures.

We describe a patient with a MM deposit in the stomach causing gastrointestinal bleeding and the need for repeated blood transfusion. Treatment was by laparoscopic wedge resection (LWR) using a combined endoscopic and laparoscopic technique. LWR of the stomach is popular technique in Japan for curative resection of early gastric adenocarcinoma[5] and there are anecdotal reports describing the use of this technique for the resection of other metastatic lesions to the stomach [6] It has many advantages compared with open surgery, especially for patients with limited life expectancy. Our aim is to highlight the use of this technique in a patient with metastatic melanoma of the stomach.

## Case presentation

**Figure 1:**
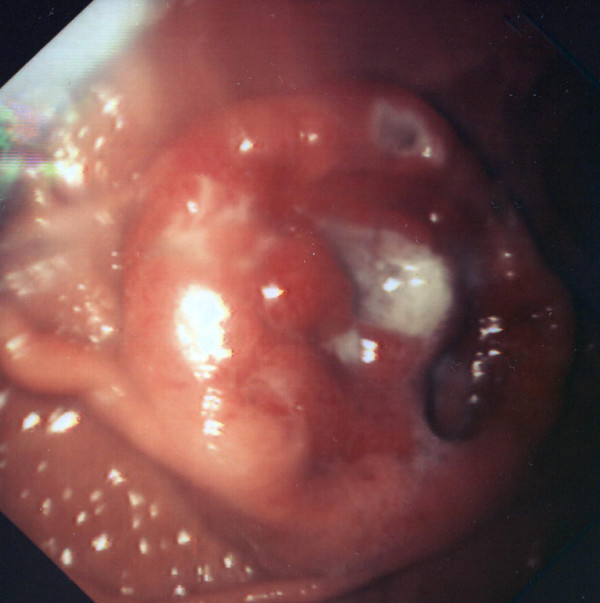
Endosopic image showing the metastatic melanoma lesion in the anterior wall of the stomach.

A 58 years old male was investigated for iron deficiency anaemia while under treatment for lung metastases from melanoma. He had been diagnosed to have cutaneous malignant melanoma 11 years previously. An oesophago-gastro-duodenoscopy (OGD) revealed a 5 cm diameter ulcer on the anterior wall of the stomach (Figure 1). There was no active bleeding, but this was felt to be the cause of his anaemia since a Computerised Tomography (CT) of the abdomen revealed no other gastrointestinal abnormality. Biopsies from the ulcer confirmed metastatic melanoma. As he was requiring repeated blood transfusions and physically fit he was offered minimally invasive resection of the stomach lesion.

**Figure 2:**
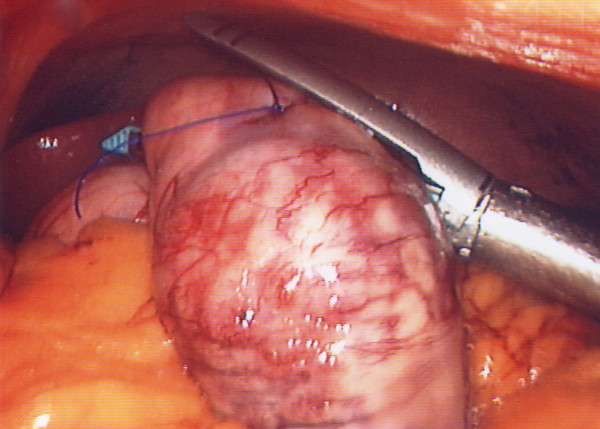
View of the forth and final firing of the Endo GIA45 stapler to complete the resection.

**Figure 3:**
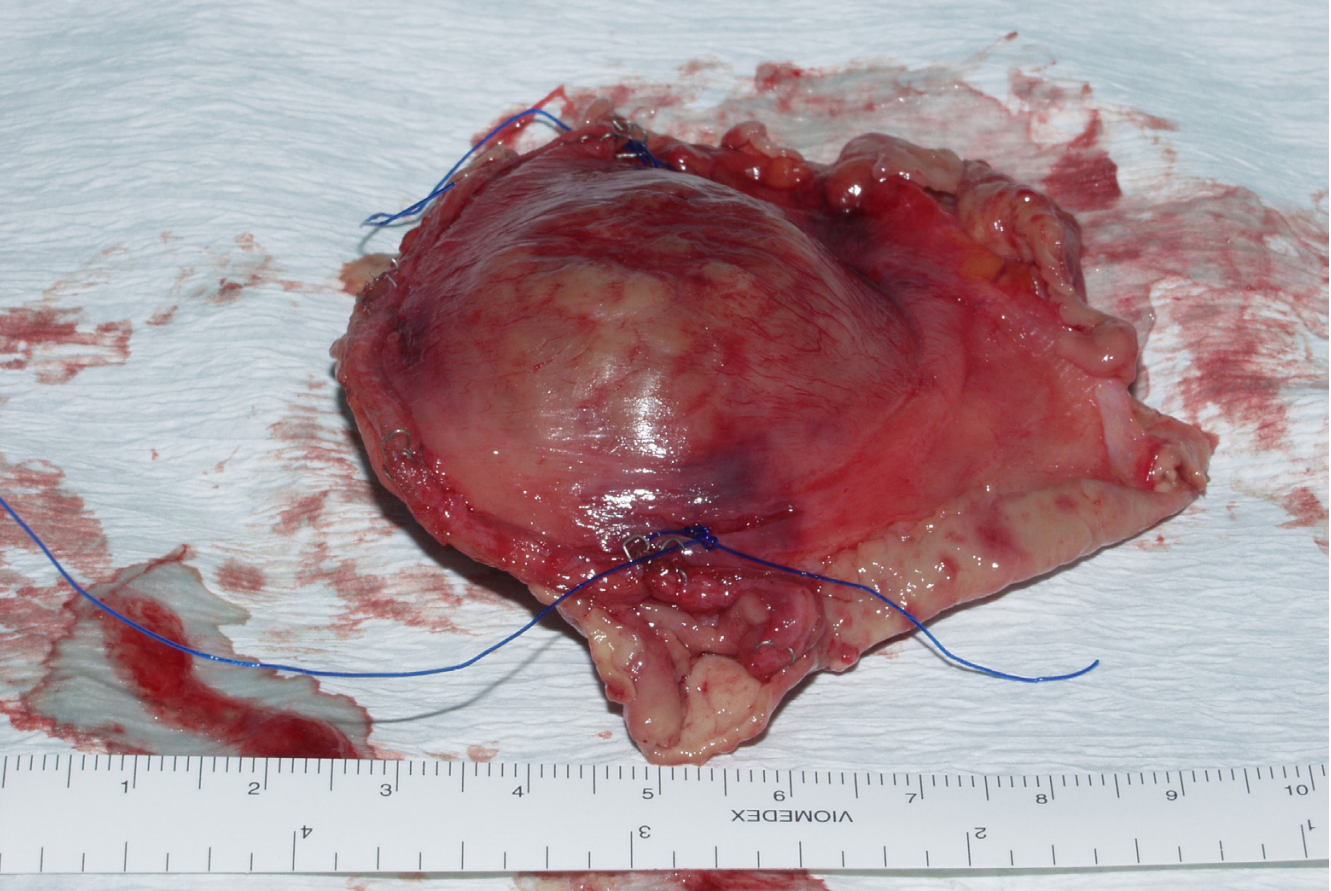
Gross macroscopic picture of the excised specimen showing adequate resection margin.

Under general anaesthesia an OGD was performed to confirm the position of the tumour and to exclude other metastatic deposits. At laparoscopy the tumour was seen on the serosal aspect of the anterior gastric body. It was elevated using two prolene stay sutures and was resected with serial applications of an endoGIA45 stapler (Ethicon Endo-surgery Inc., Cincinnati, OH, USA) to exclude the tumour (Figure 2). The staple line was over sewn using a 3-0 polypropylene suture (Ethicon Inc., Cincinnati, OH, USA). The specimen was retrieved from the abdomen in a bag. At the end of the procedure the integrity of gastric staple line and completeness of resection were confirmed by OGD. The patient made an uneventful recovery and was discharged home 48 hours after the operation. Histology of the specimen confirmed metastatic malignant melanoma measuring 35 mm in maximum dimension (Figure 3). Histology also verified a complete excision with tumour free resection margins.

The patient was well when reviewed in outpatients 8 weeks following surgery. There has been no recurrence of his anaemia and further blood transfusions have not been required.

## Discussion

Autopsy studies have shown that gastrointestinal metastases are common in patients dying of MM [7]. However, these must remain largely asymptomatic as clinical presentation during life is uncommon and occurs in only 2–4% of patients with MM [3]. Common sites of intra-abdominal metastases included small bowel (35–67%), colon (9–15%) and the stomach (5–7%)[3]. The presenting complaints in patients who have stomach metastases include symptoms of anaemia, gastrointestinal bleeding, and abdominal pain [1]. Presentation may also be as an emergency with massive bleeding, gastric outlet obstruction or perforation.

The typical feature of melanoma metastases at gastroscopy is a hemispherical submucosal nodule with central ulceration resembling a "Doughnut" [8], as in our case (Figure 1). Other possible features include multiple nodules, large extrinsic tumour mass, ulcerative or polypoid mass lesion [8]. The lesions may be pigmented or amelanotic [8]. If contrast angiography is performed during acute bleeding the classical appearance is a Bull's eye sign, but this is observed in less than 50% of the patients [9].

The laparoscopic resection of MM metastases to adrenal gland [11], gallbladder [12] and gastrocolic ligament [13] have been described. The criteria for LWR of the stomach are tumour size up to 50 mm located on the on lesser or greater curve or on the anterior aspect of the body of the stomach [10]. Tumours near the pylorus and cardia are not suitable for LWR [5]. Intra-operative gastroscopy is useful for accurate localisation of the tumour and to reconfirm the size and feasibility of the procedure [14]. It is also useful to assess completeness of resection at the end of the procedure.

Preservation of quality of life is important in the treatment of patients with gastrointestinal metastasis from MM who often have a limited life expectancy. Laparoscopic wedge resection is relatively simple technique and offers advantages of laparoscopic surgery including short hospital stay, early return to normal activity and minimal morbidity and mortality. However, care should be taken in avoiding specimen contact with the laparoscopic port sites as port site recurrence has been reported [15]. The indications for this technique may be extended to the palliative resection of rare gastric tumours in selected patients.

## Competing interests

The author(s) declare that they have no competing interest.

## Authors' contributions

**RSD: **Obtained patient consent, organised clinical photographs and wrote first draft of the manuscript. **EAG: **Performed literature search, retrieved articles and helped with manuscript preparation. **SAP: **Examined surgical specimen, took the photograph of the specimen and helped with manuscript preparation. **IMcLW: **Edited the manuscript and had overall responsibility for patient care.
